# Case of Inflamed Vein and Extensive Phlegmonous Erysipelas of the Arm, Following Venesection, Treated by Incisions

**Published:** 1828-01

**Authors:** 

**Affiliations:** St. Bartholomew's Hospital.


*Case of Inflamed Vein and extensive Phlegmonous Erysipelas of the Arm, following Venesection, treated by Incisions.* Under the care of Mr. Earle, at St. Bartholomew's Hospital.

Margaret Gearing, æt. twenty-four, a maid servant, presented herself as a casualty patient on the 28th of July, 1827. She stated that she laboured under a suppression of the catamenial discharge, and requested to lose blood, from which she always experienced immediate relief. She was bled by the dresser, and no more was heard of her till the afternoon of the 30th, two days after venesection had been performed: she then stated that, on the evening of the 29th, her arm became uneasy, which induced her to remove the bandage, and, upon examining the wound, she found it somewhat festered, and painful to the touch. She applied a poultice, and took a little opening medicine. On the 30th, she felt exceedingly unwell, her arm becoming more painful, and she begged to be admitted into the hospital.

The following symptoms were observed:—The arm, about the elbow joint, is a little swollen and painful on pressure; the wound in the vein is open, and contains a small quantity of pus; the vein is also thickened around the puncture, and upwards to the extent of half an inch; red lines, extending in the course of the absorbents, can be distinctly seen. Her countenance is anxious; skin hot and dry; pulse 100, and hard; tongue furred; bowels somewhat confined.

V.S. ad ℥xvj. (which produced syncope.)—Hyd. Submur. gr. iij.; Pulv. Antimon. gr. v. statim.—Mist. Salin. cum Mag. Sulph. ʒj. quartis horis. —Cataplasma panis brachio.

July 31st, at eight A.M.—She was seen by Mr. EARLE. The arm was now greatly swollen; erysipelatous inflammation extending from six inches above the elbow joint to the wrist; the integuments were tense and exceedingly painful. She complained of pain extending towards the axilla, the glands of which were tender to the touch. The constitutional symptoms are aggravated; pulse 100, rather more compressible. Blood drawn yesterday not buffed.

Mr. Earle stated that, unless the inflammation and tension of the limb were soon relieved, extensive sloughing of the cellular membrane would, in all probability, ensue. With the view of preventing an occurrence so likely to endanger the life of the patient, he immediately made two incisions, of about three inches in length, along the upper part of the forearm, extending down its radial side: these incisions extended down to the fascia; the cellular membrane did not appear to have yet undergone any change, but was exceedingly vascular, and cut crisp. About ten ounces of blood flowed from these incisions in the space of two hours. She expressed herself as being much relieved. Tension of the limb somewhat subsided, nor is it so tender to the touch.

The arm to be well fomented.—Continue the Saline Mixture, with Sulph. Magnesiæ.

August 1st.—She had passed a more comfortable night: pain, tension, and redness of the limb has entirely subsided. The constitutional symptoms are, however, yet severe: pulse ninety-six, and hard; tongue furred, with much pain in the head; skin rather hot; bowels inclined to be costive.

V.S. ad ℥xij.—Haust. Sennæ statim.

2d.—Feels much relieved. The arm is now of the natural size. The blood drawn yesterday was buffed.

Ordered Mist. Salin. cum Potassæ Nit. gr. viij. quartis horis.

Ten P.M.—She feels very uneasy; there is increased heat about the carpal extremity of the forearm, which is tender on pressure.

Ordered Hirudines xij. brachio. Postea Cataplasma panis.—Calomel gr. iij.; P. Antimon. gr. v. statim.—Cont^r Mist. Salin. cum Pot. Nit.

3d, eight A.M.—She is much worse. The inflammation is extending up the forearm; the integuments about the wrist and back of the hand are exceedingly tense and painful; countenance flushed; skin hot and dry; tongue furred, and bowels costive.

During the night she had a smart attack of shivering. The patient strongly urges that another incision may be made. One of four inches in length was accordingly made on the outer side of the wrist; a small quantity of pus was seen oozing out with the blood which flowed from the wound; a small portion of cellular membrane at the lower part of the wound looks in a sloughy state. About four ounces of blood were lost from this incision. The patient felt immediate relief.

The arm to be well fomented; after which, let it be covered with a bread poultice.—Calomel gr. iij.; Pulv. Jalap. gr. xv. statim.—Contin[r] Mist. Salin. cum Pot. Nit.

4th.—All unfavorable symptoms have left her. The arm is quite free from inflammation; the integuments have resumed their natural appearance. Bowels have been well opened by the Calomel and Jalap; pulse very feeble; the wounds are looking remarkably healthy.

Continue the poultice.—Ordered Inf. Cascar. ℥jss.; Tr. Card. c. ʒj. ter die.—Strong broth diet.

10th.—She is gradually regaining her strength. The two upper wounds have nearly healed; a portion of cellular tissue has sloughed away from the lower one, which in all probability is the cause of its not healing so readily as the other incisions.

Cont[r] med.

19th.—During the night she had an attack of fever, accompanied with severe pain in the head; tongue furred; skin rather hot; complains of thirst.

Applic[r] Hirud. x. temp.—Ammoniæ Subcarb. gr. xv.; Acid. Citric ℈j.; Mist. Camph. ℥jss. quartis horis, in active effervescence.

The two upper incisions have cicatrised. Another small portion of sloughy cellular membrane was removed today with a pair of forceps from the lower one.

21st.—Passed a comfortable night, and is much better.

Cont[r] med.

23d.—Not so well. Febrile symptoms have again returned; complains of nausea and headache.

Ordered Calomel gr. iij.; P. Antimon. Tart. gr. j. statim.—Mist. Salin. cum Pot. Nit. quartis horis.

24th.—The medicine has produced several copious evacuations, of a dark colour. Feels greatly relieved.

Cont[r] Mist. Salin. cum Pot. Nit.

20th.—The lower incision has now quite healed; she has perfect use of the limb. Her health rapidly improves.

Ordered Sulph. Quinine gr. ij. cum Inf. Menth. Sulph. ter die.

30th.—She was discharged quite well.

*Remarks.*—Mr. Earle considered this a well marked case of phlegmonous erysipelas, and an illustration of the good effect of incisions in the early stage of this disease. That these incisions will relieve the tension and pain of the part, and prevent the sloughing of the cellular membrane, this case alone will exemplify; for we find that where the incisions were made in the upper part of the arm, no sloughing of the cellular membrane took place, while at the lower part of the arm this process was not altogether prevented, from the incision being made at a later period of the disease: and there is little doubt but that this last incision prevented the further progress of mischief.

---

